# Resolution of Deep Venous Thrombosis: Proposed Immune Paradigms

**DOI:** 10.3390/ijms21062080

**Published:** 2020-03-18

**Authors:** J. Matthew Nicklas, Aviva E. Gordon, Peter K. Henke

**Affiliations:** School of Medicine, University of Michigan, 1500 East Medical Center Drive, Ann Arbor, MI 48109, USA; john_nicklas@brown.edu (J.M.N.); aviva.e.gordon@gmail.com (A.E.G.)

**Keywords:** venous thromboembolism, deep vein thrombosis, fibrinolysis, collagenolysis, neutrophil, stability, NETosis, macrophage, immune system, endothelial cell

## Abstract

Venous thromboembolism (VTE) is a pathology encompassing deep vein thrombosis (DVT) and pulmonary embolism (PE) associated with high morbidity and mortality. Because patients often present after a thrombus has already formed, the mechanisms that drive DVT resolution are being investigated in search of treatment. Herein, we review the current literature, including the molecular mechanisms of fibrinolysis and collagenolysis, as well as the critical cellular roles of macrophages, neutrophils, and endothelial cells. We propose two general models for the operation of the immune system in the context of venous thrombosis. In early thrombus resolution, neutrophil influx stabilizes the tissue through NETosis. Meanwhile, macrophages and intact neutrophils recognize the extracellular DNA by the TLR9 receptor and induce fibrosis, a complimentary stabilization method. At later stages of resolution, pro-inflammatory macrophages police the thrombus for pathogens, a role supported by both T-cells and mast cells. Once they verify sterility, these macrophages transform into their pro-resolving phenotype. Endothelial cells both coat the stabilized thrombus, a necessary early step, and can undergo an endothelial-mesenchymal transition, which impedes DVT resolution. Several of these interactions hold promise for future therapy.

## 1. Introduction

Venous thromboembolism (VTE) is currently one of the largest sources of morbidity and mortality, comparable in scope to Alzheimer’s disease and diabetes. This disease encompasses both deep vein thrombosis (DVT) and its potential sequelae, including pulmonary embolism (PE) if the thrombus is dislodged from the vein wall and post thrombotic syndrome (PTS) whereby the thrombus and its resolution damage the vein wall and causes venous hypertension [1]. Across recent studies (with follow-up periods ending 2009–2013) of American and European populations, the age- and sex-adjusted incidence of VTE has been reported to be 1.22–2.39 per 1000 patient-years, rising to 5–11 per 1000 patient-years by the eighth decade of life [2,3,4,5,6,7]. Of these VTE patients, 27–56% present with PE, putting them at imminent risk of death, while roughly another 20–50% of VTE patients later develop PTS, which reduces quality of life through loss in mobility, leg swelling, and skin ulcers [8].

The uncertainty regarding these complications is due in part to diagnostic inconsistencies arising from more sensitive tools such as computed tomographic angiography (CTA) [9]. Regardless, recent estimates place the 1-year mortality rate following VTE in the range of 9–30%, [3,6,10,11] compared to less than 4.5% in control patients matched for age and other major risk factors [11]. The median of these current incidence (1.57 per 1000 patient-years [5] and case-fatality rates 23%) [6,10] suggest a VTE-induced annual mortality of over 100,000 in the US population. Current treatment for VTE is primary anticoagulation, but hemostasis is impaired and many patients present with DVT that has already progressed past the point of anticoagulant efficacy [12,13]. Thus, there is potential clinical utility in investigating and controlling the process by which DVTs resolve in order to provide better care to these later-stage patients, with less bleeding risk.

## 2. Overview of Venous Thrombus Development in Humans

**Figure 1:**
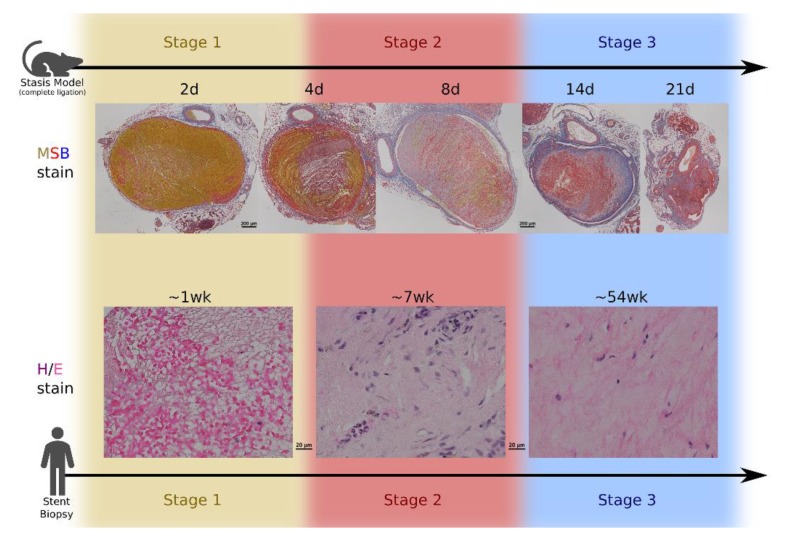
(**Top**) Histological time progression at 10× power showing venous thrombus resolution in a C57 murine IVC ligation model of DVT at 2, 4, 8, 14, and 21 days post-ligation. Formalin-fixed paraffin-embedded thrombus samples were stained using a modified Lendrum’s martius yellow, crystal scarlett, methyl blue (MSB) stain. (1) Erythrocytes are visualized in yellow, fibrin in red, and collagen in blue. As time progresses, clot composition transitions from erythrocytes to fibrin and then to collagen. (**Bottom**) Hematoxylin and eosin (H&E) staining at 100× power of human DVT paraffin-embedded tissue samples dated from venous stent implantation date. DVT stage was determined in accordance with the DVT pathology grading guide described by Fineschi et al. [16]. Within one week, the thrombus is mostly erythrocytes and platelets (stage 1), then by two months its composition shifts to include many infiltrating immune cells (stage 2), and finally the remaining tissue at the one year time point shows mostly collagen with sparse cells (stage 3). All photomicrographs were captured as stitched 4 picture mosaics using a Nikon E400 microscope and Nikon DS-Ri1 camera. The top row of photos were cropped horizontally.

The time sequence of DVT development and resolution was correctly suggested as early as 1962, based on empirical stains that detected changes in the nanoscopic roughness of clotted material [14]. As Lendrum et. al. remarked, “we consider that the intracytoplasmic granules, which we think are engulfed fibrin, seen in pulmonary phagocytes and in the endothelium of arteries and veins containing thrombus, are better shown by this (methyl-scarlet-blue) method than by any of the others.” In fresh clot (whether in deep veins or in diabetic kidneys), platelets and erythrocytes are initially linked in a fine mesh yielding a relatively smooth surface (stage 1). A mat of the medium-textured fibrin replaces the initial thrombus (stage 2), which is gradually replaced by much coarser collagen (stage 3). This staging of clot development quickly became the basis of a pathological guide to grade thrombi [15]. The current three-stage refinement of the DVT grading scheme indicates that the turnover of erythrocyte-rich to fibrin-rich clot in humans is complete approximately seven days after the initial thrombotic event [16], whereas fibrin deposition commences after one day [17]. Various leukocytes, initially neutrophils and later macrophages, are believed to control this progression of DVTs development from a mass of erythrocytes, to fibrin, and finally to collagen [18]. Infiltrating T-lymphocytes and macrophages containing hemosiderin accumulate throughout this process and then remain within the thrombus. Once fibrin predominates, endothelial cells begin recoating the thrombus. After two months, the thrombus appears hyalinized with central sinuous cavities [16], indicative of a primary collagen composition produced by fibroblasts [19]. Densely packed collagen fibers contain sparse immune cells, whereas looser tracts of collagen are associated with neovascularization [20]. Thrombus maturation in the above fashion (see Figure 1) carries the potential outcome of incorporating the collagenous post-thrombotic tissue into the vein wall and restoring patency, albeit, possibly at the cost of vein wall fibrosis and non-compliant vein walls [21,22,23].

## 3. Dependence on Murine Models

Due to the logistical difficulty of directly investigating DVT in humans, several experimental murine models of DVT have been used extensively as critically reviewed by Diaz et al. [24,25]. The two most common models involve surgical complete ligation or partial ligation (stenosis) of the mouse inferior vena cava (IVC). While the ligation model yields more consistent thrombi, partial stenosis is believed to more closely mimic the hemodynamic environment of venous valve-pockets where most DVT initiate [26,27]. These surgical models are also commonly applied to other rodents. Unfiltered PubMed searches on “venous thrombosis” and “DVT resolution” over the past 20 years revealed that 703 articles involve mice, 610 involve rats, and 250 involve rabbits. These closely related rodent models are not always in agreement, as demonstrated by the differential effects of neutropenia on DVT size in the mouse [28] and rat [29]. Other animal models of VTE are much less commonly used. In comparable PubMed searches, <190 articles describe experiments conducted on swine (primarily for testing venous stents and other devices [30]), and basic VTE research on nonhuman primates or zebrafish yielded <10 publications for each. Humans are evolutionary closer to swine than rodents, and thus genetic differences are ~30% less prevalent between porcine models and patients [31]. While genetic manipulation of these porcine models is generally more arduous than murine models, recently developed genetic tools (most notably CRISPR/Cas9) have made precise modifications feasible [32]. Regardless, mice and other rodents will remain the primary experimental VTE model for the conceivable future, as they are less expensive principally due to their small size.

There are several critical differences between human VTE and these rodent DVT models that warrant consideration. First, DVT does not occur naturally in rodents, and hence must be induced by a surgical model. Second resolution in the mouse or rat is typically measured by directly assessing the physical and histological characteristics of the thrombosed IVC, which are taken as surrogates for the clinically relevant outcomes of PE and PTS. However, roughly 40% of fatal PE events in humans may not show evidence of the original location from which the thrombus embolized [33]. Since PE events are rarely monitored in murine models of VTE [34], the absence of thrombus in the IVC can be incorrectly interpreted as thrombus resolution rather than embolization. Third, the surgical creation of these experimental models closely mimics DVT as 4a complication to major surgery or traumatic injury [35], but may induce a very different global immune response when compared to those induced by pure immobility [36,37]. Fourth, there is no component of venous hypertension in these common mouse models, although it can be simulated by creation of an arteriovenous fistula [38]. In contrast, chronic venous hypertension from venous reflux has a cross-sectional prevalence of roughly 20% in humans, although this estimate varies greatly with population and testing methods [39,40,41]. Fifth, there are significant biochemical and cellular differences in the innate immune system of mice compared to humans [42,43]. Most notably, mouse blood has a higher lymphocyte: neutrophil ratio than humans (3–9:1 versus 0.5–1:1), [44] and a higher platelet count (by 3–5 fold) [45,46]. Compared to humans, murine immune cells exhibit opposite circadian trafficking, involving both CXCR4 expression and p38 mitogen-activated protein kinases’ response [47]. Both the use of humanized mouse models and comparison of proteomic networks [48] could alleviate the risk that immune-related mechanisms of thrombus resolution operate unexpectedly in human patients. However, these novel stem cell and computational approaches have not been widely deployed in the murine models of VTE. In particular, the authors of this review highly suggest adapting the experimental techniques of Zhao et al. [47] to reconstitute the immune system of experimental DVT model mice via radiation of pups and subsequent transplantation with human CD34^+^ cells from a variety of VTE patients. Despite the potential for these differences between human patients and rodent models to confound molecular results, immunohistology time series demonstrate that the pattern of pathophysiological tissue remodeling is very similar [49].

## 4. Immune Cells: Attack, then Repair

Although antibiotics have significantly attenuated this risk, sepsis continues to pose a major health threat and results in a US mortality of 200,000 deaths per year [50]. Multiple lines of evidence indicate that the same mechanisms which protect against infection can also play a role in the formation of DVT. As early as 1952, fibrin was shown to be an effective physical barrier against foreign particles [51]. DVT can be initiated by damage to the endothelial cells lining the vein wall [52] at the site of a potential infection, as well as with lipopolysaccharide endotoxemia from Gram-negative bacteremia [53], and even with colonization of the small bowel with Gram-negative bacteria [54]. Thus, thrombus represents an effective response to sepsis both because it has the potential to envelop and contain the point source of an infection, and also because it is the concentrated precipitate of filters that remove pathogens from blood [55].

Immune cells infiltrating a thrombus may by default be programmed to assume that it contains foreign material, including pathogens, and mount a full immune response. Only once the immune system establishes that a thrombus is sterile does the resolution progress, and this requires additional detection of the thrombus microenvironment to take the appropriate next step. Both policing and repairing phases are mediated by various cytokines and chemokines [56]. Biochemically or genetically shifting the immune response in ways that simulate sepsis or hypersensitize it toward infection slow thrombus resolution because immune cells cannot remodel the tissue until they recognize that their primary job of sterilization has been achieved. Hypothetically, desensitizing immune cells to infection may augment thrombus resolution, but carries the risk of impairing a systemic immune response to a septic insult. Supplanting a particular type of engineered or selected immune cell against a septic antigen that mediates thrombus resolution is a promising potential therapy. For instance, engineered CAR-T cells yielded a significant reduction in cardiac fibrosis and could be investigated for their potential regarding VTE [57].

## 5. Neutrophils and the Potential Regulation of Early Structural Stability

An important immune mechanism linked to DVT formation is that of NETosis. Neutrophil extracellular traps (NETs), or extracellular chromatin released by self-destructing neutrophils (polymorphonuclear neutrophils, or PMNs), ensnare invading bacteria [58]. This fine-webbing also acts as a scaffold onto which erythrocytes and platelets can aggregate, thereby contributing to early clot formation [59,60]. Among other things, activated platelets expressing High Mobility Group Box 1 (HMGB1) can independently trigger: the recruitment of PMNs to the site of the developing thrombus, NET formation, and monocyte infiltration [61,62]. NETosis can also be triggered by strong cellular forces, such as high pressure gradients [63]. Immunoglobulin from patients with antiphospholipid syndrome also triggers NETosis and amplifies thrombosis [64].

Neutrophils are among the first immune cells to infiltrate the thrombus, initially outnumbering monocyte-derived macrophages (Mo/MΦ) by 7:1 at 24 h and then steadily decreasing in number by roughly 50% each week [65]. They are recruited by the neutrophil chemoattractant IL8, which both increases fibrosis and decreases thrombus size [66]. PMNs possess the ability to degrade fibrinogen internally, potentially giving them the ability to initially infiltrate the forming thrombus environment that could overwhelm other cell types [67]. Neutropenia in rats yields larger thrombi and increased collagen deposition and keratinocyte cytokine [29]. Similarly, in human patients, low PMN counts in relation to monocytes confers an increased risk for DVT [68]. Reduction of PMN also creates a more severe vein wall injury, while reducing urokinase-type plasminogen activator (uPA) and matrix metalloprotease 9 (MMP-9) and increasing MMP-2, [69] suggesting a role in later vein wall response to injury. Thrombus size increases but vein wall elasticity and collagen remain normal if neutropenia is induced 24 h after thrombogenesis.

Resolvin D4, an anti-inflammatory lipid mediator, reduces PMN infiltration and antagonizes NET formation, in addition to recruiting pro-resolving monocytes and reducing the thrombus size [70]. Furthermore, both toll-like receptor 4 (TLR4), an immune receptor, and intercellular adhesion molecule 1 (ICAM-1) respond to lipopolysaccharide binding, but reducing PMN counts increases thrombosis and increases plasminogen activator inhibitor-1 (PAI-1), increases circulating Factor XIII, and decreases uPA observed in endotoxemia [71]. This contradicts a prior result showing no neutropenic change on thrombus size in mice. Additionally, the CXCR2 chemokine receptor, primarily expressed on PMN but also on monocytes, displays a knock-out phenotype vaguely similar to neutropenia (larger thrombus, fewer PMNs and monocytes, more fibrin, reduced uPA, increased MMP-9 and reduced MMP-2) [28].

NET-related extracellular DNA is recognized by the toll-like receptor 9 (TLR9) on both monocytes and PMNs, [72,73] and a mutation in this receptor has been linked to VTE in human patients [74,75]. TLR9^-/-^ mouse VT models have demonstrated a consistent pattern of enlarged thrombus, reductions in collagen and fibrin deposition, and a dramatic increase in PMNs at 2 days and Mo/MΦ cells after 1 week [76]. The wild-type thrombus phenotype is restored by transferring the TLR9^+/+^ bone marrow-derived monocytes into a TLR9^-/-^ mouse, although this does not rescue vein wall fibrosis [77]. This suggests that TLR9 chiefly allows Mo/MΦ cells to recognize the ‘remains’ of NETs and then dispose of them. Although a subsequent study in a partial stenosis mouse model only trended non-significantly toward development of larger thrombus size in the absence of NETs, it demonstrates that citrullinated histones, a marker of NETs, are elevated in TLR9^-/-^ mice [78]. Moreover, PMN depletion in TLR9^-/-^ mice, following 2 days of experimental stasis, yields thrombi that are larger than those observed in wild type mice but smaller than those formed in TLR9^-/-^ mice.

**Figure 2:**
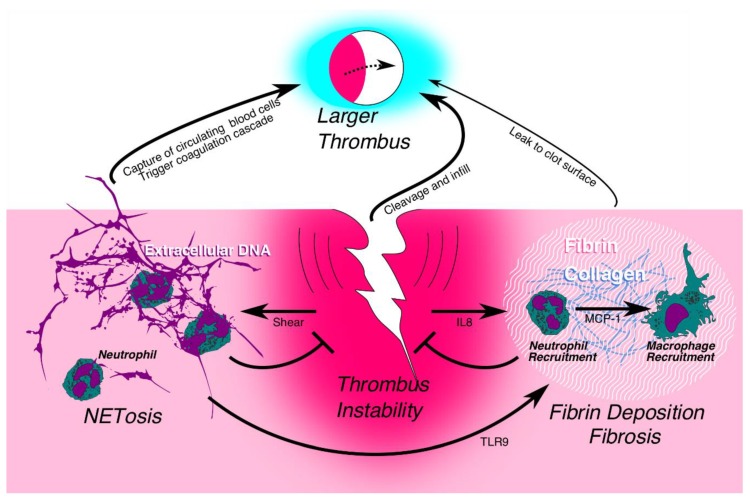
Proposed mechanism of early thrombus resolution involving neutrophils (PMNs). NETosis and fibrosis are distinct mechanisms for increasing thrombus stability and may prevent embolization. NETs do not play an early role in complete stasis thrombus, but do in non-stasis models. Whether thrombus instability without NETs leads to embolization is speculative. However, it is likely that TLR9 signaling is important for later thrombus resolution, in part by driving monocyte fibrin metabolism and clearance of sterile breakdown products. Neutropenia in TLR9^-/-^ mice impairs both NETosis and fibrosis, resulting in an intermediate thrombus size because of instability alone. This figure also illustrates a potential mechanism of immune cell influx into the developing thrombus: neutrophils are first recruited by the chemoattractant IL8, then these neutrophils trigger an influx of monocyte-derived macrophages, partially through MCP-1 signaling.

In light of this abundance of evidence, we hypothesize the following early resolution mechanism (Figure 2). The major influx of PMNs within the first day of DVT development may serve to stabilize the thrombus via a NET-like matrix. Mo/MΦ cells subsequently recognize this extracellular chromatin via the TLR9 receptor (and possibly other receptors), phagocytize it, and concurrently deposit fibrin. In the context of neutropenia or NET inhibition, in a complete stasis model, the lack of NET matrix may result in a larger VT, as well as a more fibrotic phenotype (more fibrin and collagen) as Mo/MΦ cells establish structural integrity. The same NET inhibition experiment in a partial stenosis model may result in an unstable thrombus and impaired thrombogenesis. Late responses of Mo/MΦ have not been examined in the stenosis model in TLR9^-/-^ mice.

## 6. Monocyte-Derived Macrophages and Thrombus Resolution

Monocyte-derived macrophages (Mo/MΦ) are perhaps the most important effectors of the two-phase immune-directed thrombus resolution [79,80,81,82]. They gradually infiltrate the thrombus throughout the first week of development, after which the thrombus begins to shrink in size [18,65]. Monocytes are recruited into the thrombus by monocyte chemotactic protein-1 (MCP-1, also known as CCL2) [83]. Within models of bacterial infection, MCP-1 recruitment of monocytes was dramatically enhanced by the presence of earlier-arriving neutrophils [84], which can even produce their own MCP-1 [85]. Mo/MΦ are among the most common cells within the thrombus during stages 2 and 3 of resolution [16,65]. Interestingly, elevated monocyte counts in routine blood tests (as well as a more varied distribution of red blood cell sizes) are linked to nearly 3-fold higher risks of DVT [86]. Injections of peritoneal macrophages decreased experimental thrombus size by 5-fold and MCP-1 decreased size by 6-fold [87], which are among the largest positive effects on DVT resolution observed to date. The discovery of a spectrum of activation patterns for Mo/MΦ cells, with extremes now designated M1 or pro-inflammatory and M2 or pro-resolving [88,89,90], has allowed researchers to begin deciphering this immune control.

Subpopulations of monocytes (originally found in the blood and lymph nodes, but not in the lung, thymus, and breast milk) can rapidly cocoon themselves with fibrin deposits [91], and crosslink that fibrin by secreting Factor XIIIa, a function with pathologic implications ranging from skin wound healing [92] to lung cancer [93]. In the context of pathologic DVT in human patients, there is a polarization toward pro-inflammatory Mo/MΦ as sampled from the blood [94]. These pro-inflammatory human Mo/MΦ cells are characterized by greater IL6, TNFα, and cellular adhesion markers (such as ICAM-1) expression. There is a plethora of other markers for pro-inflammatory Mo/MΦ cells, several of which differ between humans and mice [89,90,95]. Although gene expression is broadly similar, 95% of circulating human monocytes are pro-inflammatory as identified by CD16^−^, compared to 50% of mouse monocytes that are pro-inflammatory as identified by Ly6C^hi^ [96]. Additionally, markers of pro-inflammatory Mo/MΦ include IL-1β, IL12, IL8, MCP-1, CD80, and CD11c [89,97]. Furthermore, there is significant confusion arising from differential expression of surface markers in vivo and in vitro exposure to interferon gamma (IFNγ) or lipopolysaccharide (LPS) [90], as many genes are not regulated in the same way in these two situations [98]. However, in general, pro-inflammatory Mo/MΦ cells are functionally specialized to kill pathogenic, infected, or cancerous cells via phagocytosis or reactive oxygen and nitrogen species [97,99]. Directly depleting mice of LysM^+^ cells improves thrombus resolution in mice, and knocking out the transcription factor T-bet (linked to M1 activation) has the same effect [100]. Within IFNγ-deficient mice, thrombus resolution is likewise accelerated [101].

Therefore, pro-inflammatory Mo/MΦ cells fill the proposed policing role within the DVT immune system paradigm. As similar to professional police, they are poised to both block flow and assail threats, and remodeling cannot begin until they finish their ‘immunologic investigation’. Within a sterile clot, the number of policing cells is inversely tied to the pace of DVT resolution, because it takes time to ‘convince’ the pro-inflammatory Mo/MΦ to ‘stand down’ and transform into pro-healing Mo/MΦ.

Mo/MΦ cells also directly contribute to the process of DVT resolution through a variety of mechanisms associated with the pro-resolving state. They phagocytize erythrocytes, platelets, matrix debris, and the cellular remains from apoptosis, such as NETs which likely contribute to stage 1 clot stability but delay resolution [61,102,103,104]. Pro-resolving Mo/MΦ cells directly express fibrinolytic and collagenolytic enzymes that allow them to invade the thrombus tissue, particularly uPA and MMP9 [105,106,107,108]. After pro-resolving Mo/MΦ cells burrow tunnels through fibrin and collagen, it has been shown in other disease models that fibroblasts backfill these paths with collagen [107,109]. Neovascularization or angiogenesis is also promoted by pro-resolving Mo/MΦ cells [28,110,111], and although evidence is mixed whether neovascularization directly speeds DVT resolution [112,113], it is associated with reduced collagen density in PTS [20]. The majority of Mo/MΦ cells within experimental thrombus models demonstrate pro-resolving expression patterns [114], particularly IL4, IL10, CD206, CD163, VEGF, and Arg-1 markers [89,97,115]. Exogenous administration of IL10 accelerates thrombus resolution by decreasing inflammation [116]. While larger and older thrombi are associated with an overabundance of pro-resolving Mo/MΦ cells [114,117], this may be because greater effort is required to resolve a larger thrombus. Consistently, depletion of pro-resolving Mo/MΦ cells significantly impairs DVT resolution [118]. As a reflection of the activity of pro-resolving Mo/MΦ cells, IL10 levels are significantly elevated in DVT and PTS patients, but in non-thrombotic patients, high IL10 levels appear protective [119,120]. Conversely, lowering the ratio of pro-inflammatory to pro-resolving Mo/MΦ cells via bone marrow stem cell transplantation in rabbits increases the speed of thrombus resolution [121]. IFNγ-deficient mice displayed a phenotype characteristic of pro-resolving Mo/MΦ activation, with enhanced MM9 and VGEF expression [101]. The pro-resolving Mo/MΦ state can likely be activated by hepatocyte growth factor (HGF) as well, although this has not yet been reported in a VTE model [122].

In human patients, IL6 is predictive of DVT [119,120]. In mice, IL6 is expressed on both pro-inflammatory and pro-resolving Mo/MΦ cells via different mechanisms [89,123,124]. This may explain the lack of a significant difference in thrombus size at 2 days between control and IL-6^-/-^ mice, [21] as both policing and reparative Mo/MΦ function is somewhat impaired. IL-6^-/-^ mice showed impaired resolution at later time points as measured by weaker blood flow and increased 5-day thrombus size [125]. Mice treated with anti-IL6 antibodies have impaired monocyte influx and improved DVT resolution, an effect that was tied to reduced chemokine expression, particularly of MCP-1 [126]. The growth arrest-specific 6 (GAS6) signaling protein inhibits IL6, TNFα, and the nuclear factor kappa B (NF-κB) in Mo/MΦ cells [127]. Knockout mice for GAS6 have reduced monocyte recruitment, as well as less expression of both MCP-1 and CCR2 (a chemokine receptor) [128]. CCR2 appears to be critical for monocytes to exit the bone marrow and enter the developing thrombus [87,129].

The source of these Mo/MΦ cells and any implications for DVT resolution remain uncertain [80]. While the descendant monocytes of bone-marrow derived endothelial progenitors are deployed to the developing venous thrombus [130], there exist splenic monocyte reservoirs that also participate in vascular inflammation [131]. Additionally, tissue-resident macrophages may be able to self-renew, and could behave differently than Mo/MΦ cells [80,132]. Pro-resolving Mo/MΦ cells are purported to arise from pro-inflammatory Mo/MΦ cells within skeletal muscle and diabetic wounds [133,134]. Consistent with this proposed pattern, pro-inflammatory Mo/MΦ cells tend to be short-lived and rapidly recruited to sites of inflammation, whereas pro-resolving Mo/MΦ cells patrol noninflamed tissues over prolonged periods [135,136].

**Figure 3:**
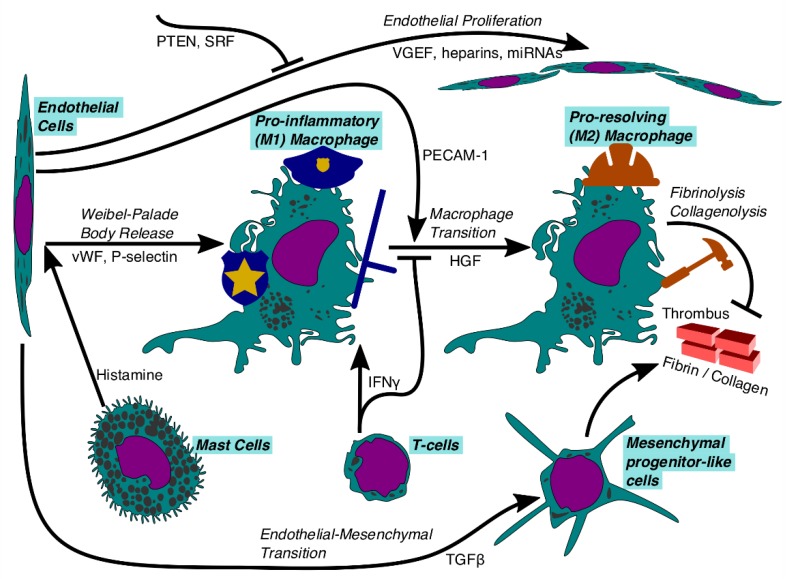
Global mechanism of thrombus resolution via some of the immune system cells. The top row of cells are ambivalent or beneficial to DVT resolution, whereas the bottom row of cells impede DVT resolution. Endothelial cells can either aid in thrombus resolution by proliferating to seal off the developing thrombus, or transition into mesenchymal progenitor-like cells, which slow thrombus resolution by depositing more collagen. Both mast cells and T-cells trigger the activation of pro-inflammatory macrophages, with mast cells potentially using endothelial cells as intermediaries. T-cells secrete interferon gamma (IFNγ) which locks macrophages into the pro-inflammatory phenotype. However, with PECAM-1 from endothelial cells or hepatocyte growth factor (HGF), pro-inflammatory macrophages transition to become pro-resolving, at which point they promote fibrinolysis and collagenolysis.

Additional immune cells serve to adjust the pro-inflammatory/pro-resolving Mo/MΦ polarization in the context of developing DVT (see Figure 3), a role consistent with macrophages being a much earlier evolutionarily development than other immune cells [97]. For instance, T-lymphocytes appear to infiltrate the thrombus early in its development [65]. These largely effector-memory T-cells produce IFNγ even when no antigens are present, thereby triggering some early inflammation [137]. However, if the T-cells recognize antigens, secretion of IFNγ increases dramatically [138]. IFNγ completely suppresses the pro-resolving Mo/MΦ phenotype and converts these cells back to pro-inflammatory expression [139]. Thus, T-cells serve as a safety-check on thrombus sterility, but their elimination speeds resolution [137]. Similarly, tissue-resident mast cells are critical to rapidly recognizing and responding to potential immune threats, including allergens and DVT [140]. Depletion of mast cells in an experimental mouse model confers protection from DVT, as the immune system activation is impaired: mast cell histamine fails to trigger endothelial cell release of Wiebel–Palade bodies containing von Willebrand’s factor and the adhesion proteins P-selectin and ICAM-1, which are implicated in monocyte recruitment [141]. This mast cell sensitivity explains the observed link between allergy and VTE [142].

## 7. Endothelial Cells

Thrombus resolution also involves endothelial cells, which assume many roles within the developing thrombus, similar to macrophages. Some of the effects of proteins secreted by endothelial cells can be understood by their linkage to the aforementioned parts of the immune system, such as ICAM-1 which is also expressed by PMNs [71] and pro-inflammatory Mo/MΦ cells [94], and Vascular Endothelial Growth Factor (VEGF) which triggers pro-resolving Mo/MΦ activation [89,101]. As noted above, endothelial cells can act as intermediaries between mast cells and infiltrating immune cells [141].

Cell adhesion modules, which were initially understood in the context of venous thrombogenesis [143], are also significant predictors for post-thrombotic resolution [120,144,145] These include P-selectin [146,147,148] and E-selectin [149,150], which are receptors on endothelial cells that specifically bind and activate immune cells in early thrombogenesis and are elevated in acute DVT. Inhibition of these selectins both prophylactically and as a post-thrombotic treatment improved DVT resolution. In contrast, the absence of another cell-to-cell signaling molecule, platelet endothelial cell adhesion molecule 1 (PECAM-1 or CD31), [151] inhibits thrombus resolution. PECAM-1 fulfills a variety of roles in thrombosis, including several that are pro-thrombotic and pro-inflammatory: platelet adhesion and aggregation, allowing immune cells to adhere to and cross the endothelial membrane, and transducing high shear stress into inflammatory signals [152]. PECAM-1 can change conformation, and thus, it can also act in more important anti-inflammatory roles: suppression of Mo/MΦ cells’ pro-inflammatory phenotype and inflammatory cytokines, sealing the endothelial vascular wall at endothelial cell junctions, and preventing apoptosis of endothelial cells [153,154].

On a cellular level, endothelial progenitor cells accelerate DVT resolution [155], because the thrombus must be covered with new endothelium as it progresses to stage 2 within the first week [156], a process regulated by a vast array of molecules. Endothelial proliferation and migration is promoted by VGEF, angiopoietins, bone morphogenic proteins [157], endothelins [158], heparins [159], and various endogenous micro-RNAs [160,161,162,163]. Homocysteine [164], phosphatase and tensin homolog (PTEN) [160], the tumor necrosis factor ligand FASLG, [161] SRC kinase signaling inhibitor 1 (SRCIN1) [162], and serum response factor (SRF) [163] delay re-endothelialization, which may have therapeutic utility by lengthening the window of opportunity for use of common anticoagulants, or thrombolytic agents.

Another significant endothelial cell transformation in the context of DVT is the endothelial-mesenchymal transition (EndMT), in which endothelial cells transdifferentiate into mesenchymal-like cells, an essential process in embryonic heart development [165]. Within a developing DVT, cells that have undergone EndMT no longer express endothelial proteins such as PECAM-1, and instead produce proteins such as α-smooth muscle actin and both Type I and Type III collagen [166]. Unsurprisingly, EndMT may impair VT resolution, as the overall pro-resolving endothelial cells are replaced with fibrotic mesenchymal-like cells. This process undergoes positive feedback because more severe thrombosis also triggers more extensive EndMT, as observed in chimeric iliac vein compression models [167]. EndMT is triggered primarily by extracellular transforming growth factor-β (TGFβ), and this signal is transduced through endothelin-1 expression [168]. It can also be activated by monocyte chemoattractant protein-2 (MCP-2, also known as CCL8), which is under positive regulation by NF-κB [169].

## 8. Fibrinolysis

As the best understood aspect of venous thrombus resolution, the enzymatic disintegration of fibrin bears discussion. This process begins to occur during stage 2 of DVT development. In mice, roughly 55% of clot area is observed to be fibrin and 35% collagen (around the periphery) at 2 weeks post-ligation. By week 4, only 20% of the thrombus area is fibrin (mostly granular patches within the interior), with collagen comprising the rest [170]. Surprisingly, there remains disagreement regarding whether neutrophils and macrophages are more numerous within the murine thrombus over this late time period [65,170,171]. The liver-secreted zymogen plasminogen, following activation and conversion to plasmin, degrades fibrin [172] into an E-fragment and D-dimer [173]. This serine protease and its three primary activators were known by the mid-1950s [174], and the specific proteolytic activation site (R^561^-V^562^) was identified in 1967 [175]. Streptokinase and the closely related staphylokinase are secreted by bacterial pathogens to allow them to escape ‘immunothrombotic’ containment [55,176,177], but are not normally involved in DVT resolution. Tissue plasminogen activator (tPA), retained on the surface of endothelial cells [178], appears essential to typical thrombus resolution as evidenced by both blood values in the context of DVT and genetics [179,180,181]. uPA is efficient at resolving thrombi in murine models [182,183], possibly because it is secreted by both endothelial cells and monocytes as they are infiltrating the thrombus and differentiating into macrophages [105,184], which in turn promotes additional circulating monocyte recruitment and differentiation as the thrombus matures [185,186].

Several proteins can also inhibit the fibrinolytic activity of plasmin directly or indirectly. Within circulating blood, α_2_-antiplasmin (α2AP) rapidly binds to plasmin, thereby halting fibrinolysis [187,188]. Deficiencies in α2AP are linked to rare bleeding disorders and increased mortality in models of PE [189,190]. Thrombin-activatable fibrinolysis inhibitor (TAFI) operates primarily by removing the C-terminal lysine from fibrin polymers, thereby preventing several amplification mechanisms of fibrinolysis that occur when plasminogen binds to fibrin [181,191,192]. Although TAFI deficiency lacks a murine phenotype [193,194], human genetic and microscopic imaging studies demonstrate a hemostatic role [195,196]. Factor XIII, which crosslinks fibrin to render it more stable [79], also crosslinks α2AP to fibrin, where α2AP prevents fibrinolysis by serving as a shield against plasmin [197].

PAI-1 inhibits fibrinolysis by cleaving tPA and uPA via the serine protease mechanism [198]. This gene’s expression is strongly linked to VTE within large Genome Wide Association Studies (GWAS) and mice overexpressing PAI-1 form 50% larger thrombus than mice with PAI-1 deficiency [199,200,201]. However, animal studies of PAI-1 inhibition have been mixed. For instance, a low-dose of the PAI-1 inhibitor tiplaxtinin improved rodent DVT resolution, whereas an increased dose resulted in larger DVTs [202,203]. PAI-2 also cleaves tPA and uPA, but much more slowly than PAI-1 [204]. Experimental models of PAI-2 deficiency exhibit accelerated DVT resolution without significantly affecting fibrinolysis [205,206]. PAI-2 is primarily retained intercellularly where it binds misfolded proteins, but can be abundantly secreted by pro-inflammatory macrophages [207,208], and acts to regulate many aspects of circulating monocyte and macrophage behavior [209,210]. Within apolipoprotein E gene-deleted mice, hyperlipidemia resulted in a significantly elevated level of PAI-1 and an undetectable uPA concentration, so fibrinolysis and thrombus resolution were severely impaired [211]. Rosuvastatin has been shown to reduce these resolution-impairing effects in both mice and trial populations of human patients [212,213].

## 9. Collagenolysis

In the final period of DVT resolution (stage 3), permanent damage to the vein wall is believed to be a critical factor potentially leading to the development of PTS [214]. Venous segments measured from human patients with chronic PTS are over twice as thick as those in comparable controls [22], although various inflammatory markers (as mentioned below) are also strong predictors of PTS [119,215]. The terminology regarding this late stage of venous clot resolution is somewhat ambiguous. The thrombosed material is absorbed into the vascular wall, as this process creates three mechanically distinct vein wall surfaces: the entrapped wall (through which immune cells migrate to reach the thrombus), the fresh/intra-thrombus wall, and the remaining sections of original vascular wall (possibly either naïve to direct thrombus contact or formerly enveloped wall). These distinctions are emphasized by differences between mechanistic murine models of thrombosis [216]. Collagen is deposited both within the remodeling thrombus and the vein wall, peaking at 12 days in a mouse model [217]. In human PTS femoral sections, this material is >80% Type I collagen, with the remainder consisting of Type III collagen [20].

Extensive concurrent collagenolysis occurs within the resolving thrombus, especially early on within stasis models and are Types III early and later Type I [216]. Collagenolysis can be carried out interstitially by MMP and neutrophil elastase [218]. Neutrophil elastase and MMP2 appear ineffective toward Type III collagen [219,220], while MMP9 can digest both Types I and III [221]. Within human DVT patients, most of the MMP enzymes appear elevated (with the exception of MMP3) [222]. Mouse models recapitulate this elevation, and further suggest that while MMP9 which is elevated throughout DVT remodeling, MMP2 and Membrane-Type-1 MMP are further elevated at later stages [217]. The decreasing ratio of MMP9/MMP2 can be used to estimate thrombus age [223].

In contrast to fibrinolysis, and rather paradoxically, inhibition of collagenolysis yields less collagen at late DVT resolution. Mice with global MMP2^-/-^ demonstrate larger venous thrombi, decreased vein wall collagen, greater monocyte influx, and fewer von Willebrand’s factor (vWF)-tagged neovascular channels [224]. MMP9 genetic knockout mice also display impaired thrombus resolution and less collagen, plus vein walls that are significantly less compliant (more stiff) in the axial flow direction, purportedly due to the biomechanical components of both the extracellular matrix and collagen/elastin [225]. The two enzymes display compensatory expression, so the phenotype is more severe when both are inhibited [224]. Collagenolysis thus appears to an important early step as immune cells infiltrate the developing thrombus through the entrapped vascular wall. When collagenolysis is impaired, so is all of DVT resolution, resulting in fewer experimental mice reaching stage 3 when most collagen deposition occurs. In contrast, thrombus resolution is promoted by IL6 signaling, which augments MMP2, MMP9, and uPA in Mo/MΦ cells [125].

MMP2 transcription is dependent on the presence of p53, better-known for its role in tumor suppression [226]. Expression of p53 also stimulates PAI-1 secretion in endothelial cells (among other genes in endothelial cells). The effect of reducing fibrinolysis outweighs that of increasing collagenolysis, because overexpression of p53 increases thrombus size [227], and in aged mice an endothelial knockout of p53 protects against DVT [228]. However, genetic or pharmacologic inhibition of p53 impairs DVT resolution in younger adult mice, whereas the p53 agonist quinacrine accelerates this resolution [229]. Exploration of the differing roles of p53 on DVT resolution further clarify the fibrinolysis and collagenolysis balance. MMP9 is assumed to be upregulated by leptin via transcription factor activator protein-1 (AP-1) and NF-κB, cleaved into its active form by plasmin, and degraded by various tissue inhibitors of metalloproteinase [230,231]. However, because overexpression of MMPs make connective tissues weaker throughout the body, this overexpression is linked to the rupture of atherosclerotic plaques [230,232], cancer invasion [233,234], and aortic aneurysm [235].

## 10. Conclusions

Much has been learned about experimental DVT on the cellular and biochemical level and from this, we postulate the following (Table 1). Fibrinolysis and collagenolysis are both required to fully resolve and heal thrombotic tissue. Neutrophils are more important in early thrombus resolution (stage 1), where they may affect stability and protect against PE through the dual mechanisms of NETosis and fibrosis. In stage 2 of thrombus resolution, pro-inflammatory monocyte-derived macrophages infiltrate the developing thrombus and likely transition to pro-resolving Mo/MΦ if sterile. Although T-cells and mast cells promote this pro-inflammatory response, inflammation eventually gives way to pro-resolving macrophages that ‘exchange’ fibrin for collagen and retract the thrombus into the vascular wall (stage 3). Endothelial cells are implicated in a variety of activities, most notably proliferation to coat the stabilized thrombus during stage 1 and possibly undergo endothelial-mesenchymal transition. It is unclear if these pathobiological mechanisms will translate into human therapies, but there exists a vast array of potential targets, and much investigation remains to be done.

## Figures and Tables

**Table 1:** Primary cellular types and roles in venous thrombus resolution.

Cell Type	Role in VTE Resolution
**Neutrophils (PMN)**	Infiltrate the early developing thrombus, also maintain thrombus stability via NETosis and early fibrosis.
**Monocyte-derived Macrophages (Mo/MΦ)**	Pro-inflammatory macrophages are activated to police the thrombus by stabilizing it via fibrosis, searching for pathogens, and releasing inflammatory markers.
	Pro-resolving (M2) macrophages phagocytize erythrocytes, conduct both fibrinolysis and collagenolysis, and promote neovascularization.
**Mast Cells**	Trigger the activation of pro-inflammatory Mo/MΦ cells by sending inflammatory signals such as histamine to endothelial cells, causing Weibel-Palade body release.
**T-Cells**	Secrete IFNγ among other immune factors that trigger pro-inflammatory Mo/MΦ cell recruitment and prevent pro-resolving phenotype.
**Endothelial Cells**	Produce surface markers and other proteins that recruit immune cells to the site of the developing thrombus and allow them to adhere. Also proliferate to coat the developing thrombus in endothelium or transdifferentiate into mesenchymal-like cells.
